# Using Mobile Health to Support the Chronic Care Model: Developing an Institutional Initiative

**DOI:** 10.1155/2012/871925

**Published:** 2012-12-05

**Authors:** Shantanu Nundy, Jonathan J. Dick, Anna P. Goddu, Patrick Hogan, Chen-Yuan E. Lu, Marla C. Solomon, Arnell Bussie, Marshall H. Chin, Monica E. Peek

**Affiliations:** ^1^Section of General Internal Medicine, Department of Medicine, University of Chicago, Chicago, IL 60637, USA; ^2^Chicago Center for Diabetes Translation Research, University of Chicago, Chicago, IL 60637, USA; ^3^College of Physicians and Surgeons, Columbia University, New York, NY 10032, USA; ^4^Department of Pediatric Endocrinology, University of Illinois at Chicago, Chicago, IL 60612, USA; ^5^University of Chicago Health Plan, Burr Ridge, IL 60527, USA

## Abstract

*Background*. Self-management support and team-based care are essential elements of the Chronic Care Model but are often limited by staff availability and reimbursement. Mobile phones are a promising platform for improving chronic care but there are few examples of successful health system implementation. *Program Development*. An iterative process of program design was built upon a pilot study and engaged multiple institutional stakeholders. Patients identified having a “human face” to the pilot program as essential. Stakeholders recognized the need to integrate the program with primary and specialty care but voiced concerns about competing demands on clinician time. *Program Description*. Nurse administrators at a university-affiliated health plan use automated text messaging to provide personalized self-management support for member patients with diabetes and facilitate care coordination with the primary care team. For example, when a patient texts a request to meet with a dietitian, a nurse-administrator coordinates with the primary care team to provide a referral. *Conclusion*. Our innovative program enables the existing health system to support a *de novo* care management program by leveraging mobile technology. The program supports self-management and team-based care in a way that we believe engages patients yet meets the limited availability of providers and needs of health plan administrators.

## 1. Introduction

Despite the availability of effective treatments, chronic diseases are often poorly controlled and remain a leading cause of preventable morbidity and mortality and excess costs worldwide [1, 2]. Outcomes are generally worse for patients of lower socioeconomic status, including racial and ethnic minorities, who experience higher rates of disease and complications, lower quality and access to care, and limited health literacy and social support [3, 4]. 

The Chronic Care Model (CCM) has been proposed as a multicomponent intervention to improve chronic care [5, 6]. Self-management support is a cornerstone of CCM and has been linked to improved health outcomes in a range of chronic conditions [7]. Yet, in practice, self-management support programs are poorly reimbursed, difficult to scale, and often unavailable [8]. Payers and providers recognize the need for self-management support programs, but often lack the human or financial resources needed to adequately provide them [8]. Existing programs may not be suitable for racial and ethnic minorities and traditionally rely on one-on-one or group sessions, which are resource intensive [8, 9]. In addition, CCM emphasizes team-based care that engages nonphysician clinicians and offers care management for high-risk patients [10]. However, with the current emphasis on cost containment, many organizations are reluctant to implement programs that require new personnel.

Mobile technology is a promising platform for improving chronic care because it leverages a technology that most patients already use—mobile or cellular phones [11]. Unlike most technologies, mobile phones enjoy wider usage in racial and ethnic minorities and low-income groups [12] and thus have the potential to address health disparities. Through text messaging, or Short Message Service (SMS), and other mobile applications, patients and providers can communicate between standard office visits and thereby complement traditional clinic-based models of care [13]. Several studies have successfully piloted mobile phone- and text messaging-based programs in asthma, obesity, smoking, and diabetes [14], and a recent clinical trial demonstrated improved disease control in patients with diabetes who received a mobile coaching program compared to usual care [15]. 

While these results are promising, experience with mobile health chronic care programs outside of research settings is surprisingly limited. Many mobile health interventions may not be appropriate for real-world health systems. For example, many interventions use mobile phones primarily as telemonitoring devices (e.g., to collect glucose measurements or blood pressure readings) and send alerts to providers whenever abnormal readings are detected. These programs risk overburdening providers [16] and may have low rates of patient adherence even in research settings [17, 18]. Furthermore, little is known about how to design mobile health interventions that integrate with and strengthen existing systems of chronic care [19]. 

The outstanding questions are significant: how can the interventions be funded; who should be responsible for their administration; how can patients be efficiently enrolled and monitored; what content best engages patients; what information should be collected; and, crucially, how to effectively integrate into existing clinical workflows. In this paper, we report on our experience developing a text message-based diabetes program at the University of Chicago. We comment on lessons learned from our collaboration with clinicians, university administrators, researchers, and implementation personnel and describe a *de novo* care management program that is funded and operated by a university-affiliated health plan.

## 2. Program Setting

University of Chicago Medicine (UCM) is a large academic medical center on Chicago's South Side serving a predominantly urban, working class African-American community. The medical center includes an acute care hospital and a comprehensive ambulatory care center including an internal medicine practice (the Primary Care Group (PCG)) and endocrinology practice with diabetes expertise (the Kovler Diabetes Center (Kovler)). UCM largely functions as a fee-for-service provider. Outpatient care is provided in traditional office-based settings with physicians providing the vast majority of care and nurses and ancillary staff providing basic triage. Currently, diabetes self-management education (DMSE) is largely provided through individual clinic appointments with physicians or other members of the diabetes team (e.g., diabetes educators, nutritionists); outside of research settings, diabetes classes or formal DMSE programs are not available.

The University of Chicago Health Plan (UCHP) is a 10,000-member health plan largely comprised of employees of the University or the Medical Center and their dependents. Eighty-five percent of service utilization is at UCM, which charges UCHP on a fee-for-service basis, with the balance provided out of network. UCHP largely functions as a traditional health plan and prior to the study operated no disease management programs or care management services. UCHP employs two registered nurses (RNs) who function primarily in an administrative capacity.

UCHP members include university faculty and administrators, students and resident trainees, and ancillary staff (e.g., maintenance, food services) who largely reside in the urban, working class African-American community surrounding the medical center. The average age of enrollees is 40 years of age, and fewer than 10 percent are over age 60. Fifty-three percent of medical center beneficiaries and 18% of university beneficiaries are African-American. 

Approximately 380 UCHP members have type 1 or type 2 diabetes. Of these, roughly one-third of patients have well-controlled diabetes (hemoglobin A1c or HbA1c below 7%), one-third are moderately controlled (HbA1c between 7% and 8%), and one-third are poorly controlled (HbA1c above 8%).

## 3. Program Development

We utilized an iterative process of program development that built upon a prior pilot study and engaged key institutional stakeholders. 

### 3.1. Pilot Study and Behavioral Research

In January 2010, we completed a four-week pilot study that provided automated self-management reminders to African-Americans with diabetes. This study investigated the acceptability and feasibility of mobile phone-based interventions in our patient population. The results demonstrated high levels of patient engagement and satisfaction, along with preliminary evidence of improvements in self-management [20]. 

A key lesson was the importance of having a “human face” to the text messaging program. Participants reported that they needed “somebody somewhere” to be involved with the texting program to maintain their interest. During enrollment, participants met in-person with a member of the research team, who then called participants weekly to monitor their experience. Although the role of this staff member was intended for data collection, many patients identified her as part of the intervention and felt that the social connection they developed with her facilitated their engagement in the texting program. 

More broadly, the pilot study demonstrated that the effects of the text messaging program went beyond automated reminders. In-depth analysis of one-on-one participant interviews revealed that the program modified participants' beliefs about their diabetes, increased their confidence in self-management, and most surprisingly, provided social support—findings that contributed to a new behavioral model of how mobile phone-based interventions affect self-management [21]. This behavioral model informed the further development of our diabetes text messaging program. 

### 3.2. Content Development

Pilot participants suggested a number of improvements including varying message content from week to week, providing more control to adjust message timing and frequency, and adding additional content about nutrition and physical activity. Based on these recommendations, a multidisciplinary team of physicians, diabetes educators, and researchers developed a bank of over 500 text messages. The text messages cover a range of diabetes self-management topics including medication taking, glucose monitoring, nutrition, physical activity, and foot care as well as topics relevant to living with any chronic illness (Table 1). Messages are organized into various message types including *education* (to modify health beliefs), *prompts* (to perform a behavior), *tips* (to facilitate adoption of a behavior), *encouragement* (to increase perceived support), *assessments* (to collect self-reported behaviors), and *feedback* (to reinforce positive and negative behavior). The language, style, and format of the messages were largely adapted from our pilot study in which participants created their own messages. Messages were written at an eighth grade reading level and piloted internally to assess comprehension. As our study population is almost entirely English-speaking, the messages were developed in English only.

### 3.3. Stakeholder Engagement

During program development, we regularly sought the advice and recommendations of many distinct stakeholders: primary care physicians and endocrinologists; nurses and diabetes educators; university and medical center administrators; and diabetes and health disparities researchers. The process leveraged the institutional support and infrastructure established by Improving Diabetes Care and Outcomes on the South Side of Chicago, a multifactorial intervention with a quality improvement collaborative of six clinics including PCG and Kovler [22]. Between May 2011 and March 2012, our team held several meetings and conference calls until a common vision emerged. The following sections outline key contributions of the various stakeholders.

**Clinicians**

Physicians and nurses welcomed the addition of a diabetes program that would provide between-visit support to patients on a regular basis. No concerns were expressed about loss of control over patient care, and instead many eagerly sought a team-based approach to self-management support. Although clinicians supported the program, they felt it would not be feasible for the clinic to administer the text messaging program. The clinic had recently undergone a difficult electronic health record (EMR) implementation, and neither physicians nor nurses had the time to enroll and monitor patients on the texting system. At the same time, they voiced concerns about how the program would manage clinically relevant text messages, such as low or high blood sugar readings. With clinician input, we devised protocols to escalate care in response to incoming patient text messages.

**Primary Care Group (PCG) and Kovler Diabetes Center (Kovler) Leadership**

Clinic leaders were enthusiastic about the concept behind the program because they recognized the widespread need for self-management support interventions and the challenges of traditional office-based approaches. However, concerns were raised about how best to engage providers given their limited availability and the fact the text message software would be distinct from the clinic EMR. Given these concerns, the program was designed such that clinicians would not be required to directly interface with the text messaging software. Rather, they would only be required to respond to emails from program administrators about clinical issues, as email is already part of clinic workflow.

**University of Chicago Health Plan (UCHP)**

A senior administrator at UCHP was impressed by the high levels of patient satisfaction with the pilot study and believed that a largely automated program would be feasible for the health plan to implement. She embraced the opportunity to provide regular support to beneficiaries with chronic illnesses and increase member satisfaction. However, such a project would only be possible if it did not require significant new costs or personnel. UCHP had the capacity of 0.25–0.50 FTEs to dedicate to this project. Coincidentally, two RN-trained administrators were seeking opportunities to leverage their nursing training and interact more directly with patients. UCHP required that these administrators not provide direct clinical care, such as telephone-based triage or medication titration. Instead, their focus would be on enrolling and monitoring members on the texting software and coordinating care with the primary care team. The legal team was also concerned about HIPAA compliance given that text messaging is not considered a HIPAA-secure form of communication. At the same time, the large-scale use of mobile health was unfamiliar territory for the institution and a significant evaluation component was needed. The decision was made to implement the program initially as a research study with Institutional Review Board approval. In addition, to minimize HIPAA concerns, text messages were written with no protected health information and, whenever possible, without naming diabetes or specific medications that could link the patient to the disease.

## 4. Program Description

**Figure 1:**
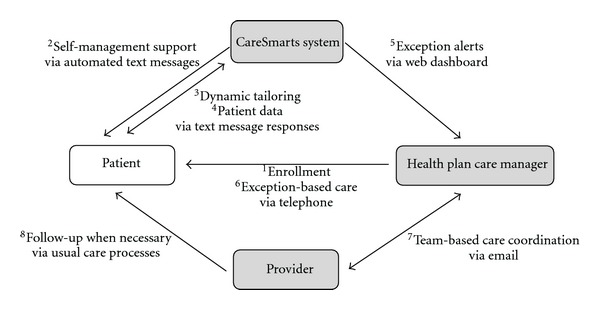
Conceptual representation of the text message-based chronic care program. ^1^Health plan care managers enroll eligible member with type 1 or type 2 diabetes over the phone. ^2^Patient receives self-management support via automated text messages and respond to queries via text message. ^3^Patient's responses help to dynamically tailor content. ^4^The CareSmarts system collects patient data from the text message responses and ^5^alerts the care manager when exceptions require attention. ^6^Using protocols, the care manager provides telephone-based support to the patient and ^7^coordinates care with the provider. ^8^The provider follows up with the patient for any necessary care.

We developed a text message-based diabetes program that provides self-management support and team-based care management for patients with diabetes (Figure 1). The program is fully funded by UCHP and is offered free of charge to members with type 1 or type 2 diabetes who receive care at the University of Chicago Medicine (UCM). Each participant is enrolled in the program for a period of six months, after which UCHP will determine whether to extend the program. UCHP contracted with a software vendor mHealth Solutions, LLC (New York, NY) for a mobile health software program called CareSmarts. The following is an overview of the key features of this project.

### 4.1. Program Administration

The University of Chicago Health Plan serves as the primary site of the diabetes text messaging program. UCHP RN-trained administrators (henceforth referred to as “care managers”) enroll eligible members in the program, monitor system-generated alerts, and coordinate care with the primary and subspecialty care teams. 

### 4.2. Eligibility

Members are eligible if they are English-speaking adults ages 18 and older and carry the diagnosis of type 1 or type 2 diabetes. Exclusion criteria include members who are not their own primary caregiver, live in a nursing home, or do not have access to a personal mobile phone. Physician consent is obtained prior to recruitment and physicians have the option to exclude individual patients or their entire panel. Enrollment in other DMSE programs or diabetes research studies is not an exclusion criterion and is in fact encouraged.

### 4.3. Enrollment

UCHP care managers enroll eligible members over the phone (Figure 1, step 1). Enrollment is completely voluntary and members have the opportunity to leave the program at any time. The enrollment process takes between 15 and 20 minutes. While it would have been feasible for patients to enroll themselves online, care managers personally enroll patients to circumvent any patient barriers to Internet access and to foster a personal relationship with each patient. To further build rapport, patients are provided a web link to a YouTube video in which each care manager briefly introduces herself and explains why she is excited about participating in the program. 

### 4.4. CareSmarts System Tutorial

Following enrollment, participants begin exchanging text messages with the CareSmarts system through their personal mobile phones (Figure 1, step 2). During the first week, participants are trained on how to use the system. They are taught to respond to questions using a particular syntax (e.g., yes or no for yes/no questions or 1, 2, or 3 for multiple choice questions) and to respond within 1 hour of receiving of the query. When a patient responds in a form the system cannot interpret, she receives a follow-up text message to facilitate learning. For example, “We did not understand your response—in the future please only respond to yes/no questions with either a yes or no.” Nonresponses are logged and at the end of the tutorial week participants with less than an 80 percent response rate are contacted by phone to troubleshoot technical difficulties and encourage adherence. After the first week, an alert is generated for participants with low response rates, which prompts the care manager to reach out to the patient and reengage them in the program. Throughout the program, patients are reminded that the system is not an emergency response system and to only send messages in response to queries.

### 4.5. Self-Management Support

Text messages exchanged with patients are organized into four domains: education, medication reminders, glucose monitoring reminders, and foot care reminders. Each domain is comprised of two-week modules, which vary by topic and frequency of messages. Each two-week education module focuses on one diabetes self-management topic: medications, nutrition, glucose monitoring, foot care, or exercise. In addition, each module covers a specific topic relevant to living with a chronic illness such as navigating the health care system or coping with stress. The other three domains—medication reminders, glucose monitoring reminders, and foot care reminders—are designed to support behavior change. They consist largely of *reminders* (“Time to take your diabetes medication.”), *tips* (“Keep your medications next to the sink so they become part of your morning routine.”), *assessments* (“On how many of the last seven days did you take all of your diabetes medications?”), and *feedback* (“Great job!”). Medication reminders are sent up to twice a day based on the time participants indicate they prefer to take their medications and the frequency with which they want reminders.

### 4.6. Dynamic Tailoring

The modules patients receive in each domain are tailored every two weeks based on their preferences and ongoing interactions with the text messaging system (Figure 1, step 3). During enrollment, care managers use a web-based dashboard to enter information about a patient's diabetes medication and glucose monitoring regimen, self-management behaviors, and message timing preferences, which are used to personalize the initial content of the program. Patients set preferences about the times of day they would like to receive reminder messages, based on the timing of their morning and evening doses of medications and their personal and work schedules on weekdays and weekends. Patients who do not take any medications for diabetes (e.g., diet-controlled diabetes) do not receive medication messages; similarly, those who are not prescribed glucose monitoring do not receive any glucose monitoring messages. Subsequently, every two weeks, the system queries patients about their preferences on the content of the texting program and collects self-reported adherence information (Figure 1, step 4). For example, it will ask, “On how many of the past 7 days did you take all of your diabetes medications?” Patients' responses are used to enroll patients in a new set of modules for the following two weeks. For the education domain, patient preferences largely drive enrollment; for the three reminder domains, enrollment is based on patient preferences about the timing of reminders and self-reported adherence. The number of text messages participants receive varies based on the modules they are enrolled in and their individual preferences, but on average participants are expected to receive 2-3 messages per day for the first half of the program and 0-1 messages per day in the second half of the program.

### 4.7. Exception Alerts

Certain patient responses to queries trigger an alert to the care manager (Figure 1, step 5). These messages are exceptions, defined as a patient response requiring the attention of the care manager. The care managers have protocols to respond to each type of exception alert and are expected to respond by the next business day (e.g., within 24 hours during the weekday or the following Monday during weekend). For example, if a participant reports suboptimal medication adherence during the weekly adherence assessment, an alert is triggered. Upon logging into the web-based dashboard, the care manager is notified of a new alert requiring her attention. Per protocol, care managers call the patient and administer a structured assessment to understand the reason(s) for the patient's low medication adherence (Figure 1, step 6). This information is then communicated to the primary care and endocrinology teams over email (Figure 1, step 7). For patients only seen in primary care, emails are sent to directly to their primary care physicians; for those seen in both primary care and endocrinology, emails are sent to an email account shared by a team of Kovler diabetes educators and the primary care physician is copied. Using existing clinical workflows, providers then decide the appropriate followup; this may include telephone reassurance, referrals to diabetes educators or dietitian, or scheduling an appointment (Figure 1, step 8). Queries are sent to participants on all days of the week and at various times, depending on participants' timing preferences, and so alerts are also generated throughout the week. As care managers are only available Monday through Friday during usual business hours 8am to 5pm, exception alerts are not designed to identify clinically urgent events, which would require immediate followup.

### 4.8. Care Management

The automated and integrated nature of our program fosters care management, which, per CCM, we define as an intervention creating proactive, prepared care teams. Because our program is largely automated, the care managers are only responsible for enrolling patients and responding to alerts. Care managers focus on patients who need assistance and are able to manage a large panel of patients with limited time. Through alerts, the program helps to identify patients who need additional support. Because this information is immediately shared with providers via email, it facilitates between-visit care. The program is designed to monitor and support self-management rather than provide clinical care: this matches the skill sets of the care managers and allows clinicians to maintain an arm's length involvement with the text messaging program. 

## 5. Discussion

We report on the development of a novel intervention that leverages mobile technology to provide self-management support and enhance team-based care. Our process included reflection on lessons learned and patient input from a pilot study, as well as iterative discussions with institutional stakeholders including physicians, nurses, clinic leaders, and health plan administrators. We set out to address several key issues: the need for a “human face” to the patient experience, limited clinician time, and the availability and interest of health plan staff to provide nonclinical support. The result is a widely supported initiative, funded by our University's health plan, which integrates into primary and specialty care and allows existing health plan staff to manage a population of patients with diabetes.

Central to our model is the health plan, which is staffing and financing the program. We estimate that this model requires 1 full-time-equivalent (FTE) care manager per 300 enrollees and costs $25/enrollee/month for software licensing fees and other technology-related expenses. These costs compare favorably to other care management programs reported in the literature, which typically serve 30–100 patients per FTE. From the provider perspective, our model is readily scalable. It is designed for typical outpatient practices and clinic workflows and requires minimal clinic redesign. 

The major innovation of this program is how it leverages mobile technology to enable *existing* health system resources to support the Chronic Care Model. Because the system is largely automated and designed for self-management support, dedicated staff members are not required and part-time nurse administrators are able to enroll and monitor patients. Because these care managers are first to respond to exception alerts, clinicians do not need to interface with the text messaging system and only engage with the program through direct communications with the care managers. Finally, because the program leverages a widely available mobile platform, it requires minimal upfront costs and can be provided free-to-charge to patients with a modest per-member-per-month technology fee.

Our model offers several advantages to existing care management programs and self-management interventions. First, the system is “high-touch” and engages patients on a daily basis, whereas traditional programs involve only weekly outbound calls or bimonthly classes. Second, the program is delivered through the mobile phone. Patients unable to attend classes in-person are thus able to participate, and the program is accessible wherever a patient happens to be. The program also reduces the upfront costs of implementation because it leverages this existing technology platform. Third, unlike traditional programs, our program is largely automated and thus requires less staff time. Fourth, because the program emphasizes self-management rather than clinical care, less staff training is required. While our care managers are trained as RNs, their responsibilities could be met by diabetes educators, MAs, or health coaches with additional training in diabetes self-management support. In contrast, traditional care management programs require RN or nurse practitioner training. This translates to significant cost savings: our program would cost $600/enrollee/year with a nurse-care manager compared to $415/enrollee/year with an MA—or 30 percent less [23].

Our model has two important limitations. First, we benefited from a relatively closed health system in which all of the primary and specialty care providers were part of the same institution, which facilitated program integration. However, using email communication to engage providers should be replicable in health systems in which payers and providers are less closely aligned. The growing trend towards accountable care organizations, in which payers and providers take responsibility for the health of a defined population of patients [24], may further enable diffusion of our model [25]. Second, we required patients to own a mobile phone and incur the cost of incoming and outgoing text messages. This requirement was informed by widespread availability of text messaging, including in racial and ethnic minorities and low-income groups [12]. However, in patient populations with limited access to mobile devices, the program may need to provide cell phones or reimburse the cost of text messaging, which we anticipate would increase the monthly cost of the program by an additional $10/enrollee/month. Ability to text message is less of a concern; in our prior pilot study, even patients with little to no experience with text messaging at enrollment were able to learn quickly and found the system easy to use [20].

## 6. Conclusion

Mobile health technologies hold significant promise in improving the delivery of chronic care, particularly for racial and ethnic minorities and low-income patients. However, mobile technology is a platform and not a solution in itself: only by integrating mobile health into a comprehensive chronic care model can we effectively and sustainably improve population health. Because we designed our program with systematic input from patients, providers, and health plan administrators, we believe our model is sustainable and scalable in our institution, is highly engaging for patients, and enhances self-management support and team-based care.

While we have successfully developed a promising program, its direct outcomes are not yet known. Future analyses will allow us to assess the rate of patient adoption and adherence to the texting program and the effectiveness, scalability, and sustainability of our model.

Our model may be replicable in other health systems, particularly those transitioning to accountable care organizations or which provide care management or population health services. Our approach to improving population health offers important lessons for achieving the triple aim of better health, improved health care, and lower costs with health information technologies.

## Figures and Tables

**Table 1:** Representative text messages across various topics and message types. Note that all five message types are used for each topic area. This table gives examples of some of these types for each topic.

Topic	Message type	Example text message
Medication	Education	To get the most out of your medicines, you need to take them as prescribed and on time, every day. Even if you are not feeling sick.
	Prompt	Reminder: Time for your medicine!
	Feedback	Reminder: Think about the last time you didn't take your medications. What happened? Think about what you can do to prevent it from happening again.
	Assessment	In the last 7 days how many days did you take all of your diabetes medications?

Glucose Monitoring	Education	A good blood sugar within two hours after eating is less than 180 mg/dL. A good fasting (before breakfast) blood sugar is 80 to 125 mg/dL
	Encouragement	Monitoring blood sugars is not just so your doctor knows how you are doing. Glucose monitoring is a tool for YOU to know how you are doing.
	Feedback	7 for 7, perfect job! (in response to self-report of perfect adherence to glucose monitoring)

Nutrition	Education	Corn and potatoes may be vegetables, but they are also starches that can increase your blood sugar. Stick to non-starchy vegetables like spinach and carrots.
	Tip	If it's not in your kitchen, you probably won't eat it. Avoid temptation by not keeping desserts or unhealthy snacks in the house.
	Encouragement	Developing a tasty but healthy food plan with diabetes can be hard. Diabetes educators can help. Do you want to meet with one? (yes/no)

Foot Care	Education	Increased blood sugars can cause nerve damage to the foot and decrease circulation. Over time this can lead to pain, infection, and other foot problems.
	Prompt	Reminder: Check your feet every day. You should look between the toes and bottoms of your feet for cuts, cracks, or anything else out of the usual.
	Tip	Reminder: Make a daily foot check part of your routine. Do it as you step out of the shower or when you take your shoes off at the end of the day.

Exercise	Education	Did you know that experts recommend moderate physical activity for at least 30 minutes 4 times per week? (yes/no)
	Tip	Lifting small weights at home or while you jog can build muscle and lower your blood sugars. No weights? Use a can of vegetables!
	Encouragement	It doesn't take hours of sweat-soaked exercise to get the benefits of exercise, even short period of gentle exercise can do a lot!

Living with a Chronic Illness	Education “Health Navigation” module	Tip: Your clinic has a way to see urgent patients the same day or within 48 hours. When you are not feeling well, the emergency room is not your only option.
	Education “Dealing with Stress”	Tip: Did you know that stress increases your blood sugars? In fact, not only can stress increase your sugars but high sugars can also increase your stress.
	Encouragement “Social Support”	Tip: Everyone feels bad about their diabetes from time to time, even if they have had it for a while. It's what you do with those feelings that count.
